# Inactive or moderately active human promoters are enriched for inter-individual epialleles

**DOI:** 10.1186/gb-2013-14-5-r43

**Published:** 2013-05-25

**Authors:** Carolina Gemma, Sreeram V Ramagopalan, Thomas A Down, Huriya Beyan, Mohammed I Hawa, Michelle L Holland, Paul J Hurd, Gavin Giovannoni, R David Leslie, George C Ebers, Vardhman K Rakyan

**Affiliations:** 1 The Blizard Institute, Barts and The London School of Medicine and Dentistry, Queen Mary University of London, 4 Newark Street, London E1 2AT, UK; 2 Department of Physiology, Anatomy and Genetics and Medical Research Council Functional Genomics Unit, South Parks Road, Oxford, OX1 3PT, UK; 3 The Gurdon Institute and Department of Genetics, University of Cambridge, Tennis Court Road, Cambridge CB2 1QN, UK; 4 School of Biological and Chemical Sciences, Queen Mary University of London, London, E1 4NS, UK; 5 Wellcome Trust Centre for Human Genetics, University of Oxford, Headington, Oxford OX3 7BN, UK

**Keywords:** Epigenetics, DNA methylation, epialleles

## Abstract

**Background:**

Inter-individual epigenetic variation, due to genetic, environmental or random influences, is observed in many eukaryotic species. In mammals, however, the molecular nature of epiallelic variation has been poorly defined, partly due to the restricted focus on DNA methylation. Here we report the first genome-scale investigation of mammalian epialleles that integrates genomic, methylomic, transcriptomic and histone state information.

**Results:**

First, in a small sample set, we demonstrate that non-genetically determined inter-individual differentially methylated regions (iiDMRs) can be temporally stable over at least 2 years. Then, we show that iiDMRs are associated with changes in chromatin state as measured by inter-individual differences in histone variant H2A.Z levels. However, the correlation of promoter iiDMRs with gene expression is negligible and not improved by integrating H2A.Z information. We find that most promoter epialleles, whether genetically or non-genetically determined, are associated with low levels of transcriptional activity, depleted for housekeeping genes, and either depleted for H3K4me3/enriched for H3K27me3 or lacking both these marks in human embryonic stem cells. The preferential enrichment of iiDMRs at regions of relative transcriptional inactivity validates in a larger independent cohort, and is reminiscent of observations previously made for promoters that undergo hypermethylation in various cancers, *in vitro *cell culture and ageing.

**Conclusions:**

Our work identifies potential key features of epiallelic variation in humans, including temporal stability of non-genetically determined epialleles, and concomitant perturbations of chromatin state. Furthermore, our work suggests a novel mechanistic link among inter-individual epialleles observed in the context of normal variation, cancer and ageing.

## Background

Epialleles are genomic loci at which the epigenetic state can stably vary among individuals in a given population [1]. Although first described and still best understood in plants [2-4], in recent years we have come to realise that epigenomic landscapes in mammals can also show considerable inter-individual variation (reviewed in [1,5]). Mammalian epialleles could arise through the action of *cis*- or *trans*-genetic influences [6,7], or have non-genetic origins as a result of: (1) potential stochastic events [8,9]; (2) exposure to a compromised *in utero *environment as has been shown in rodent and human studies [10-12]; (3) or adult life-style associated factors such as smoking [13]. Despite these and other previous studies, the molecular nature of mammalian epialleles, in particular those induced by non-genetic factors, has remained controversial [14]. To a large extent, this is due to the DNA methylation-focus of previous investigations [5]. Incorporation of information about the chromatin state would refine our understanding of the molecular nature and ultimately functionality of epialleles in the context of normal variation or disease states.

Here we describe the first systematic interrogation of mammalian epialleles that integrates genomic, methylomic, transcriptomic and chromatin state information. Using a combination of experimental and computational analyses we identify key features of epiallelic variation in humans, including demonstrating that even non-genetically determined epialleles can be temporally stable, and that DNA methylation variability at epialleles is associated with concomitant perturbations in chromatin state. Most notably, we find that promoter-associated epiallelic variation is predominantly associated with developmentally important and/or tissue-restricted genes. A similar category of genes is preferentially hyper-methylated in various cancers, *in vitro *cellular transformation, and human chronological ageing, potentially pointing to a novel mechanistic link between these processes and human inter-individual epiallelic variation.

## Results

### Comprehensive genomic/epigenomic/transcriptomic profiling of monozygotic (MZ) twin pairs

For the initial discovery phase of the study, we originally aimed to generate integrated genomic/epigenomic/transcriptomic profiles for CD14+ cells from three different healthy MZ twin pairs of European ancestry (MZ pair 31/32: female, age at sampling 27 years; MZ pair 21/22: female, age at sampling 27 years; MZ pair, male, 11/12, age at sampling 19 years Additional file 1, Table S2). We focussed on CD14+ cells as they can be obtained to >90% purity [17] and Additional file 1, Figure S1, and are less likely to harbor post-differentiation, random epigenetic alterations as they have a lifespan of only a few weeks. Genetic profiles were obtained using the Illumina Omni2.5S array that interrogates approximately 2.5 million single nucleotide polymorphisms (SNPs) with a minor allele frequency of down to 1%. DNA methylation was assayed by Illumina450K arrays that provide bisulfite conversion-based, single base resolution methylation measurements at approximately 450,000 different cytosines associated with a range of genomic features such as promoters, enhancers, and CpG islands (CGIs) [15]. Gene expression was profiled using the standard Illumina mRNA-seq protocol. For the analysis of chromatin state we performed ChIP-seq on the histone variant H2A.Z, which is strongly associated with transcriptional activity (but can also be found at transcriptionally silent promoters), and is thought to be environmentally responsive [16]. We obtained 60-80 million mapped 36 bp paired end reads for the ChIP-seq and RNA-seq libraries. All genomic/epigenomic/transcriptomic profiles were generated from the same single sampling of CD14+ cells for each individual. Unfortunately, during the course of processing the samples, the DNA sample that was to be used for subsequent DNA methylation analysis for individual '12' was inadvertently lost. As these twins were recruited because we had methylomic data from a previous time-point to test for temporal stability, as discussed below, it was not feasible to repeat all the functional genomic assays on additional MZ pairs.

### Identification of temporally stable inter-individual DMRs (iiDMRs)

**Figure 1:**
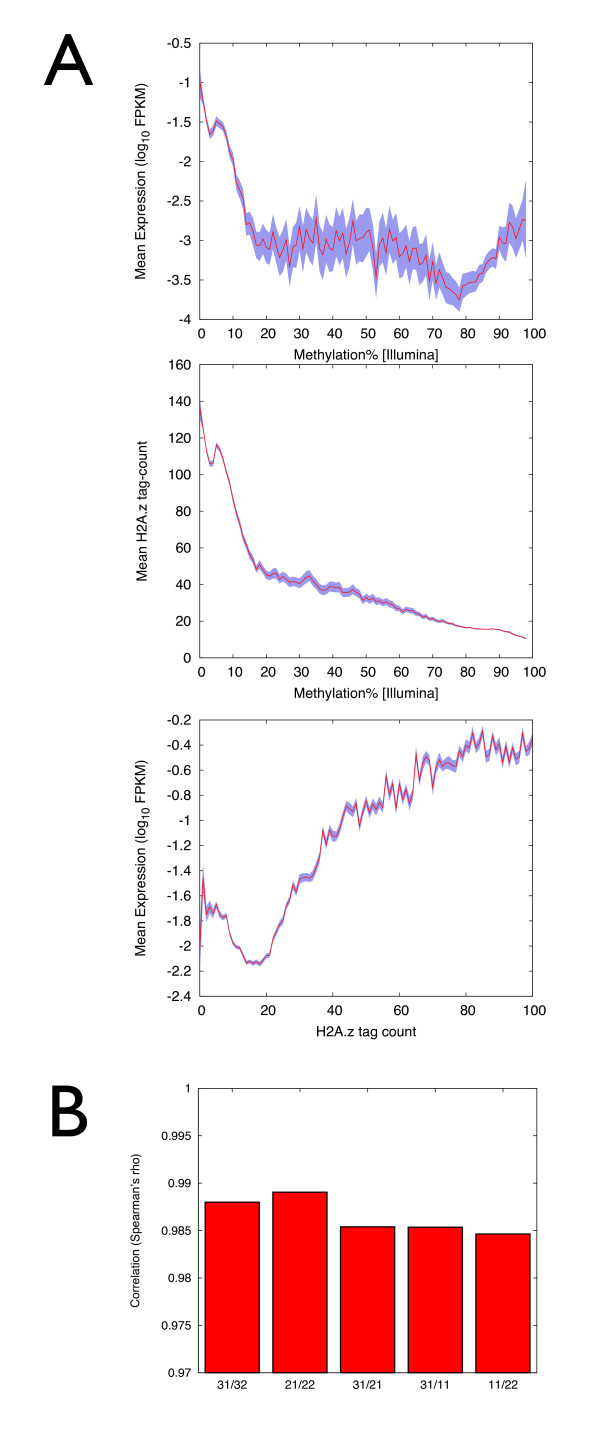
**Epigenomic, genomic and transcriptomic profiling in CD14+ cells from MZ twins**. **(A) **We analysed the following correlations at promoter regions: DNA methylation *vs*. gene expression, H2A.Z *vs*. gene expression and DNA methylation *vs*. H2A.Z. Gene expression levels are represented as log-transformed aligned average FPKM (fragments per kilobase of exon per million fragments mapped) from RNA-seq data. FPKM values were determined by TopHat and Cufflinks and assigned to TSSs. DNA methylation values were determined as β values using Illumina 450K array (see Materials and Methods) and are represented here as percentage of methylation bins. Probes on the Illumina 450K array were assigned to their closest TSS, discarding assignments >1 kb away. H2A.Z abundance is represented as normalised read counts from ChIP-seq data correlates positively with gene expression (Spearman's ρ = 0.50, *P *<10^-3^), DNA methylation is anti-correlated with H2A.Z (ρ = -0.59, *P *<10^-3^). Promoters were defined as TSS ± 1,000 bp. **(B) **Correlation coefficients of inter-individual DNA methylation differences. 31/32 and 21/22 plots correspond to MZ twin iiDMRs; 31/21, 31/11 and 11/22 correspond to iiDMRs found in unrelated individuals.

An initial low-level analysis confirmed anticipated genomic/epigenomic correlations. First, we confirmed known functional correlations between DNA methylation, H2A.Z levels, and gene expression: promoter DNA methylation was negatively correlated with both H2A.Z and gene expression levels, and promoter H2A.Z levels were strongly positively correlated with gene expression (Figure 1A). Second, analysis of the SNP arrays did not reveal intra-MZ pair SNP or copy number variation (approximately 640,000 to 660,000 SNPs observed between unrelated individuals, and 104 and 115 SNPs observed in 31/32 and 21/22 MZ pairs, respectively, and these are most likely false positives). Finally, DNA methylation profiles were substantially more similar between co-twins compared with unrelated individuals (Figure 1B).

**Figure 2:**
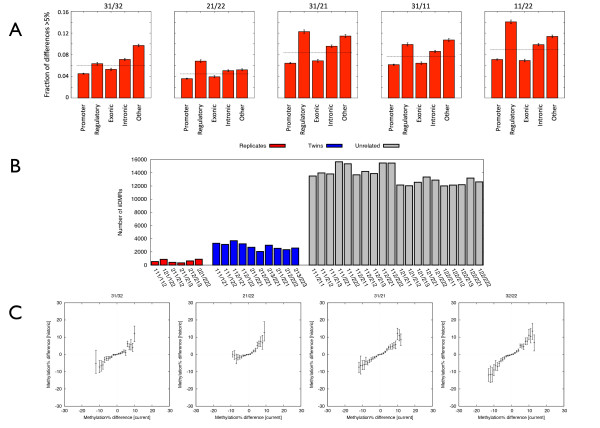
**Genomic characteristics and temporal stability of inter-individual DMRs (iiDMRs)**. **(A) **This plot shows the distribution of iiDMRs across different genomic features (promoters, regulatory regions (that is, Regulatory Features from Ensembl), exons, introns and all other regions (that is, other). iiDMRs were defined as differences >5% in DNA methylation between individuals present in two adjacent probes of the Illumina 450K array. The feint line shows the expected level if iiDMRs were found equally in all regions of the genome. **(B) **A comparison of technical *vs*. biological variation for iiDMRs. Shown is the number of iiDMR calls between Illumina 450K array for technical replicates, twin pairs and unrelated individuals. This analysis was done with CD14+ DNA from two MZ twin pairs in triplicate. The biological variation significantly exceeded the technical variation (*P *<10^-7^, permutation test). **(C) **Temporal stability of iiDMRs. The inter-individual DNA methylation differences found in this study were compared with the DNA methylation differences present in the same individuals 2 years prior. The previous DNA methylation differences were obtained from CD14+ DNA methylation profiles from the same individuals using Illumina 27K data as part of a separate study [17]. Shown are all current and historic inter-individual methylation differences from all the common probes present on Illumina 27K and Illumina 450K arrays.

We then called inter-individual DMRs (iiDMRs) between all five possible pair-wise comparisons (two different MZ pairs and one 'unpaired' co-twin). Illumina450K probe sequences were remapped using BLAT, and we only used probes that mapped exactly once. Furthermore, we ignored probes on chrX and Y, and also those that overlapped known SNPs (based on information provided in the Illumina 450K annotation file) to eliminate artefacts due to differential probe hybridization effects, resulting in a final set of 369,908 different probes. Exclusion of these probes eliminates only *cis*-acting genetic variants present in the 50 bp sequence covered by the probe, and all other *cis*- and *trans*-genetic effects are retained. We considered only those iiDMRs with a directionally consistent ≥5% absolute methylation difference at ≥2 adjacent CpGs within 500 bp of each other, as the biological relevance of very small methylation differences limited to single CpG sites is currently unclear. The number of iiDMRs found in each of the five different pairwise comparisons is shown in Table 1. Consistent with Kaminsky *et al. *[8], iiDMRs were found across the genome, but enriched at non-promoter non-exonic regions, and a greater number of iiDMRs were observed between unrelated individuals (Figure 2A). Furthermore, we generated triplicate Illumina 450K profiles for CD14+ cells from two additional MZ twin pairs, different to those described above, and found that the amount of biological variation seen between twins exceeded the technical variation (false positive iiDMR calls) by a factor of approximately 5 (*P *<10^-7^, permutation test) (Figure 2B).

**Table 1:** Number of iiDMRs identified in the various pairwise comparisons.

Comparison	iiDMRs (*n*)
31 *vs*. 32	5,077
21 *vs*. 22	2,528
31 *vs*. 21	9,512
31 *vs*. 11	9,062
11 *vs*. 22	10,526

Number of iiDMRs for each comparison was calculated as DNA methylation differences ≥5% at ≥2 adjacent CpGs within 500 bp of each other.

The epigenomic landscape for any given individual is, to an extent, constantly in flux, and many iiDMRs identified from single time-point measurements probably do not have long-term phenotypic consequences. Therefore obtaining some measure of iiDMR temporal stability is critical, especially for non-genetically determined iiDMRs. The CD14+ cells used in this study were obtained from individuals recruited in late 2010. We previously sampled the same individuals in mid-2008 as part of a separate study in which we profiled their CD14+ cells using the Illumina27K array [17]. This array contains probes for 27,578 different CpG sites, largely promoter-associated, and >90% of these CpGs are also represented on the Illumina450K array. For maximum power when comparing to the much less densely spaced probes on the Illumina27K array, we considered all >5% methylation differences, rather than just those in multiple-probe iiDMRs. Comparison of the 2010 with 2008 methylomic profiles, for CpG sites common to both platforms, revealed that a substantial proportion of the epigenetic variation seen in the 2010 samplings were also found in the 2008 samplings, that is, was temporally stable (Figure 2C). Although this is not surprising for comparisons between unrelated individuals, since many of the methylation differences in such cases most likely represent stable genetic effects, temporal stability in the intra-MZ pair comparisons is particularly noteworthy as it means that random or environmental events can induce permanent, or at least semi-permanent, epiallelic variation. Furthermore, given that the lifespan of CD14+ cells is only a few weeks, compared with the approximately 2-year gap between the first and second samplings, these epialleles most probably arise in blood progenitor cells. For the CpGs in common between the second (450K array) and first time-points (27K arrays), the proportion of methylation differences found at the second time-point that were also present at the first time-point is shown in Additional file 1, Figure S2. The number of sites where a difference in the same direction is seen at both sites significantly exceeds what would be expected by chance in all cases (*P *<3×10^-13^, χ^2 ^test). This is the first genome-scale demonstration of temporally stable epiallelic variation in mammals that cannot potentially be explained by stable genetic effects (for example, [18]). For further analyses we did not restrict ourselves to CpG sites common to the Illumina450K and 27K arrays, otherwise we would have been left with too few DMRs for any meaningful analyses, but it is reasonable to assume that the same degree of temporal stability should be present across the entire set of iiDMRs found from the Illumina450K data obtained from the 2010 samplings.

### iiDMRs at promoters anti-correlate with H2A.Z and preferentially associate with lowly expressed or silent genes

**Figure 3:**
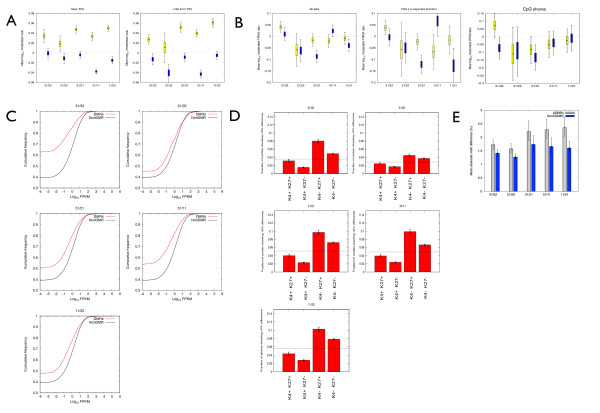
**Promoter iiDMRs anti-correlate with H2A**.Z changes and are preferentially found at genes expressed at low levels. **(A) **Anti-correlation between inter-individual DNA methylation (for only >5% methylation differences) and H2A.Z differences in CD14+ cells. Data are shown as mean and 95% credible intervals. **(B) **Inter-individual promoter epiallelic differences are not correlated with gene expression differences. For each bin of inter-individual DNA methylation differences we calculated the ratio of log transformed RNA-seq reads (FPKM) in the first individual of each comparison respect to the other. Data are represented as 95% credible intervals on the mean. The left panel shows the correlations using DNA methylation data only, the middle panel uses those epialleles in which DNA methylation and H2AZ are anti-correlated, and the right panel looks at epiallelic variation at CpG shores (as defined in the Illumina450K array annotation file). **(C) **iiDMRs are predominantly found at genes expressed at low levels. Shown is the distribution RNA-seq reads (expressed as FPKM) for iiDMRs and non-iiDMRs. In all cases the curves corresponding to DMRs are shifted to the left compared to non-DMRs showing that DMRs occur preferentially at genes expressed at low levels. For all cases 31/32 and 21/22 plots correspond to MZ twin comparisons; 31/21, 31/11 and 11/22 correspond to unrelated individuals comparisons. **(D) **Promoter iiDMRs occur preferentially at regions depleted for H3K4me3 and enriched for H3K27me3 (K4lo/K27hi) or depleted for both these marks (K4lo/K27lo) in human embryonic stem (hES) cells. hES cell H3K4me3 and H3K27me3 ChIP-seq data are from [24]. The dashed line in each plot refers to the overall iiDMR fraction against the whole dataset. **(E) **Illumina450K probes were ranked by methylation-ageing correlation using data from [20], and the 500 most- age-associated probes were taken as aDMRs, while 500 randomly selected probes from the remainder of the dataset were taken as controls. For each set, we collected absolute methylation differences from each of the five possible pairings of individuals in this study, and plot 95% credible intervals on the mean.

We next wanted to investigate whether iiDMRs are associated with altered chromatin states or gene expression, focussing on promoter-associated iiDMRs as it is currently not straightforward to correlate the activity of gene distal regulatory elements with gene expression levels. Promoters were defined as a 1 kb window centred at the annotated transcriptional start site (TSS). We observed a statistically significant anti-correlation between DNA methylation and H2A.Z at both intra- and inter-MZ pair promoter iiDMRs (Figure 3A and Additional file 1: Figure S3). This was especially marked for iiDMRs associated with >5% absolute methylation differences. Significantly, the negative H2A.Z-DNA methylation correlation was observed even at sites >1 kb away from the promoter (Figure 3A). Therefore, our data show that a significant number of iiDMRs, even those that are non-genetically determined, represent genomic sites that harbour perturbed chromatin states, not just DNA methylation differences, and thus can be classified as epialleles [19].

Interestingly, we observed only a very weak anti-correlation between promoter iiDMRs and RNA-seq gene expression levels (Figure 3B). This was true for both intra- and inter-MZ pair iiDMRs, and not significantly improved even when considering just those iiDMRs that were anti-correlated with H2A.Z, or iiDMRs with a large magnitude, or those found within CpG islands or CpG shores (Figure 3B). Not surprisingly, a direct comparison between intra-/inter-MZ pair H2A.Z variation and expression did not reveal any significant correlations either (Additional file 1, Figure S4).

The relationship between promoter DNA methylation and gene expression is known to be complex, even in contexts where large DNA methylation differences are generally observed, for example, genetically-encoded differentiation programs or cancer [5]. In our case, we found it surprising that even inclusion of H2A.Z information did not improve the strength of the correlations as, theoretically, integration of information from different components of the 'epigenetic' state of a region should yield more robust correlations. To further explore the cause for these observations, we compared mean expression levels of iiDMR promoter-associated genes with all genes in our dataset. We found promoter iiDMR-associated genes to be expressed at significantly lower levels, relative to the other genes, in all intra- and inter-MZ pair comparisons (Figure 3C). In other words, it seems that promoter-iiDMRs are associated with genes that are lowly expressed or silent in CD14+ cells.

These observations were reminiscent of previous results and our own report on human ageing-associated DNA methylation dynamics [20,21]. In those studies, human promoters that become hypermethylated with chronological age (aDMRs) were also associated with genes expressed at relatively low levels in the analysed tissue. Notably, these aDMRs were strongly enriched for promoters harbouring bivalent chromatin domains in embryonic stem (ES) cells. Bivalent domains harbour both H3K4me3, generally considered an active mark, and H3K27me3, generally considered an inactive mark [22,23]. Furthermore, bivalent domain promoters are associated with developmentally important and tissue-restricted genes [22,23]. An analysis of previously published H3K4me3 and H3K27me3 ChIP-seq profiles in human ES cells [24] revealed that promoter iiDMRs were only moderately enriched for bivalent chromatin domains (relative to genome average, refer to Figure 3D). Surprisingly though, strong statistically significant enrichment was observed for the high H3K27me3/low H3K4me3, or low H3K27me3/low H3K4me3 states in ES cells. Both these chromatin states are also strongly associated with tissue-restricted genes and indeed we found iiDMR promoters were significantly less likely to be associated with house keeping genes (*P *<10^-5 ^in all five possible pair-wise comparisons, Chi-squared test). Re-analysis of the 500 most age-correlated probes (that is, aDMRs) from [20] revealed that these probes display significantly more intra- and inter-MZ pair variability than 500 randomly selected probes (*P *<0.01, bootstrapped for all five comparisons, Figure 3E). So the common property between iiDMR and aDMR promoters seems to be a strong association with genes that are tissue-restricted, but are only moderately active, or inactive, in the analysed tissue.

### Analysis of iiDMRs in an independent cohort

**Figure 4:**
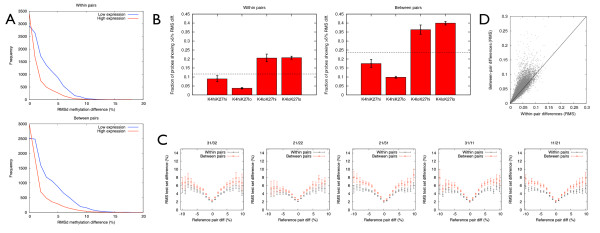
**Validation of iiDMRs in an independent cohort**. **(A) **For this analysis we used 30 MZ twin pairs from [20] whose whole blood DNA methylation profiles were generated by Illumina 27K arrays. We defined low and high expression based on the RNA-seq data we generated in this study from CD14+ cells. High expression: >1 FPKM, Low expression: <1 FPKM. **(B) **Promoter iiDMRs in the test set are preferentially enriched at regions low in H3K4me3 and high in H3K27me3, and in regions that lack both of these marks in hES cells. The analysis was performed essentially as described in the legend for Figure 3D. **(C) **Intra- and inter-MZ pair iiDMRs are correlated in the discovery and validation cohorts. For the 30 MZ pairs from [20], we measured intra-pair methylation variability by taking the RMS (root-mean-square) methylation differences for each probe. We also calculated a similar inter-pair variability measure by permuting the pairs. For each of the five possible pairs in the current study, we bin probes by methylation difference, and plot the mean inter- and intra-pair validation methylation variabilities. **(D) **For the 30 MZ pairs from [20], we calculated intra-pair and inter-pair RMS methylation variability as above and show a scatter plot for all probes on the Illumina27K array (with exclusions as described in the Methods for the Illumina450K data).

To independently validate the findings above, we re-analysed DNA methylomic data from our previously published human ageing study, that is, the test set [20]. This larger and independent dataset consists of Illumina27K methylome profiles of whole blood obtained from 30 different healthy female MZ pairs of European ancestry, ranging in age from 25 to 79 years old [20]. Although the 450K array overall has approximately 15X as many probes as the 27K, the majority of these are outside of promoter regions. Of the total annotated protein-coding genes in the human genome (21,665), 19,409 (89.6%) are associated with at least one promoter probe on the 450K array, whereas 14,400 (66.5%) are associated with at least one promoter probe on the 27K array. We calculated root mean square (RMS) differences for each probe on the Illumina27K array across the 30 different MZ pairs for both intra- and inter-MZ pair comparisons. The RMS deviation is a measure of the inter-individual methylation variability, and is directly proportional to the level of variability observed in the cohort under study. RMS difference, as opposed to mean difference, was used because the differences will be in an arbitrary direction for each pair. It is important to note that the RMS difference is not a measure of directional age-related changes. Analysis of the test set resulted in several key conclusions, applicable to both intra-MZ pair and inter-MZ pair comparisons, validating our findings from the discovery set. First, iiDMR-associated promoters found in the test set were associated with genes expressed at significantly lower levels compared with the non-iiDMR set of promoters in the CD14+ RNA-seq data generated in our study, and in a previously published whole blood array-based expression dataset (Figure 4A and Additional file 1, Figure S5). Second, the test-set iiDMRs were significantly depleted for housekeeping genes (*P *<10^-5 ^for either intra-MZ pair or inter-MZ pair comparisons, Chi-squared test). Third, iiDMRs identified in the test set were significantly enriched at promoters that harbour either high levels of H3K27me3 and low levels of H3K4me3, or are devoid of both of these marks in ES cells (Figure 4B). The fact that our findings from CD14+ cells in discovery set were validated by unsorted whole blood cell data in the test set further supports the robustness of our key conclusions. We also determined that for all five possible comparisons in the discovery dataset, there's a strong correlation of both intra- and inter-MZ pair variability between the discovery and test datasets (Figure 4C). Finally, we also asked if there is an overlap between intra- and inter-MZ pair iiDMRs. This was done using the test set since it has significantly more pairs. We found that mostly the same probes show the highest variability in both intra- and inter-MZ pair comparisons (Figure 4D). Interestingly, inter-MZ pair comparisons show bigger differences at the same sites relative to intra-pair comparisons but the great majority of these show greater variability in the between-pair comparisons (Figure 4D).

## Discussion

Our data reveal several novel and important features of mammalian epialleles. First, we find that even non-genetically determined epialleles can be temporally stable (at least over the course of 2 years). That is, a significant fraction of these epialleles are not just transient epigenetic perturbations with little prospect of influencing molecular function. Second, inter-individual DNA methylation variants are associated with perturbations of chromatin state, a relationship observed for even small differences, for example, down to approximately 5% methylation difference, and therefore can be considered as bona fide epigenetic perturbations. Of course, future studies using bigger sample numbers are needed to further explore our initial findings.

The most significant aspect of our study is the finding that the correlation of iiDMRS with gene expression differences is very weak and that iiDMRs are preferentially found in regions of relative transcriptional inactivity. So what are the implications of this? First, it is possible that some promoter epialleles show inter-related DNA methylation and chromatin state perturbations, but may not impact significantly on genome function, at least as measured by steady state transcriptional activity. In the case of non-genetically determined epialleles, maybe all promoters are potentially subject to epiallelic variation, but the more active ones are 'cleared' of aberrant epigenomic variants, whereas the less active/silent promoters can accumulate epigenetic variation. But the enrichment of epialleles in less active/silent promoters was also found in comparisons between unrelated individuals. Although it is hard to say what proportion of epialleles between unrelated individuals are due to genetic as opposed to environmental differences from our data, the genetic influence on DNA methylation profiles is well documented [3,4,25]. Bell and colleagues measured genome-wide methylation in 77 HapMap Yoruba individuals, for which gene expression and genotype data were available, and found a strong genetic component to inter-individual variation in DNA methylation profiles [4]. Although they found a significant enrichment of SNPs that affect both methylation and gene expression, they also noted that the total number of genes showing such a signal is only a small proportion of the total number of methylation variants they identified [4]. A similar conclusion was reached by Myers and colleagues who analysed genome-wide methylation in six members of a three generation family and found that only 22% of genes harbouring genotype-dependent DNA methylation exhibited allele-specific gene expression (albeit more than expected by chance) [25]. Therefore, in both cases the correlation between genetically determined DNA methylation and expression is at best modest, which would be consistent with our results regarding chromatin state.

It is possible that epiallelic variation acts in a manner not evident from simple correlations with steady-state expression levels in a given tissue. First it is possible that these correlations are tissue-restricted as has recently been shown for genetically determined tissue-restricted gene expression [26]. Alternatively, conclusions from two recent studies, although not focusing on DNA methylation/chromatin state in mammals, hint at other potential mechanisms by which epialleles could act. Yvert and colleagues recently compared H3K14 acetylation profiles between two strains of the yeast *Saccharomyces cerevisiae*, and found 5,442 sites that significantly differed in H3K14ac levels, which they called single nucleosome epi-polymorphisms (SNEPs) [27]. However, higher acetylation in one strain did not always mean higher expression of the relevant gene, for example, in one case the SNEP was associated with the strength of gene activation upon stimulation by heat shock. Secondly, Lindgren and colleagues recently assessed the effect of naturally occurring variation in miRNA expression levels on mRNA levels in humans, but found little correlation [28]. The authors concluded that their findings were more consistent with the primary role of miRNAs being to buffer mRNA levels. A key conclusion therefore is that correlating epialleles with steady-state RNA dynamics, possibly the most common analysis currently presented in papers on epiallelic investigations, may not be particularly fruitful.

Finally, and potentially most importantly, the broadly similar characteristics of iiDMRs and aDMRs (from our previous study [20] and [29]) may in fact be a general feature of mammalian epiallelic variation in a variety of contexts. Meissner and colleagues found that aberrant gradual hyper-methylation during *in vitro *cell culture is found at promoters associated with genes not expressed in that cell type [30]. Additionally, it has been found in a variety of human cancers that bivalent chromatin domains (associated with low transcriptional activity in stem cells) are preferential targets of hyper-methylation [31-33]. The common thread among these seemingly disparate examples of inter-individual epigenetic variation is promoters that are developmentally regulated and tissue-restricted, and are only moderately active, or inactive, in the analysed tissue. We propose that there could be a potentially important mechanistic link between normal/stochastic epiallelic variation and the epigenetic perturbations observed in the context of cancer and ageing.

## Conclusions

The existence of mammalian epialleles is not in doubt, but the key challenge now is to characterise epialleles at the molecular level. Our work reveals key and novel properties of epiallelic variation in humans, and further suggests important mechanistic links between normal inter-individual epigenetic variation and epigenetic perturbations observed in cancer and chronological ageing.

## Materials and methods

### Samples

Fresh venous blood was obtained from three pairs (six individuals) of healthy MZ twins (Additional file 1, Table S1). Blood was diluted 1:1 in RPMI media and then peripheral blood mononuclear cells (PBMCs) were separated by Ficoll-Hypaque gradient centrifugation. CD14+ cells were isolated according to manufacturer's instruction using magnetic bead-based positive selection system (Miltenyi Biotech). The purity of the cells was determined by FACS using CD14-FITC antibodies (Additional file 1, Figure S1). All subjects gave informed consent and the study was approved by the Northern and Yorkshire Research Ethics committee (REC Reference Number: 06-MREO-3-22). Validation of iiDMRs was done using whole blood Illumina27K data previously generated [20]. This cohort included 30 different healthy MZ female twin pairs recruited from within the UK as part of the TwinsUK registry.

### Illumina 450K array

A total of 500 ng of DNA from CD14+ cells isolated using QIAamp DNA Mini Kit was bisulfite converted using the EZ DNA Methylation kit (Zymo Research). Arrays were processed at the Barts and The London Genome Centre, London, UK according to the manufacturer's recommendations. Methylation scores for each CpG site are called as 'Beta' values (using BeadStudio software from Illumina), that range from 0 (unmethylated (U)) to 1 (fully methylated (M)) on a continuous scale, and are calculated from the intensity of the M and U alleles as the ratio of fluorescent signals.

### Illumina Omni2.5S arrays

The arrays were processed according to the manufacturer's instructions using 500 ng of DNA.

### ChIP- and RNA-seq

The chromatin immunoprecipitation (ChIP) assay was performed on 5 × 10^5 ^CD14+ cells according to previously published protocols with minor modifications [34]. Chromatin was sonicated to get fragments of 100 to 500 bp and immunoprecipitated with 10 uL of anti-H2A.Z antibody (Active Motif, Cat no: 39113). ChIP-seq libraries were prepared following the Illumina protocol and ligated to standard PE adaptors and sequenced on an Illumina GAIIx instrument. For RNA-seq, 200 ng of total RNA was used to prepare RNA-seq libraries using the TruSeq RNA kit from Illumina following the instructions provided in the supplier's manual, and sequenced on an Illumina GAIIx instrument.

### Sequence data processing

ChIP-seq reads were mapped to the GRCh37 (hg19) reference genome sequence using MAQ 0.6.6 and mappings with quality scores <10 were discarded. For iiDMR-centric analyses, we counted the numbered of paired end fragments overlapping each probe region on the Illumina array and used that as a ChIP score for that probe. RNA-seq reads were mapped to the reference genome using Tophat 1.3.1, then expression levels (FPKM) were estimated for each Ensembl transcript using Cufflinks 1.0.3. For analyses comparing methylation data to expression, methylation array probes lying within 1 kb of an Ensembl TSS were assigned an 'expression level' equal to that of the transcript associated with the nearest TSS.

### Statistical analyses

Correlation between variables was performed using Spearman's rank test. Confidence intervals for all box/bar plots are obtained by bootstrapping unless otherwise stated. Confidence intervals for the hES cell H3K4me3/H3K27me3 bar charts are estimated from a binomial model. Probes associated with housekeeping genes were defined as in [20].

For the genomic location enrichment analyses, exon, intron and regulatory features were extracted from Ensembl, and promoters were defined as regions within 1 kb of the TSSs of an Ensembl gene. For each of these categories, we asked what fraction of the probes lying in the selected regions were called as iiDMRs, and plot 95% confidence intervals on this proportion, estimated using a binomial model. For comparison, the feint line indicates the fraction of iiDMRs across the whole dataset, allowing enrichment or depletion to be assessed.

## List of abbreviations

aDMR: ageing-associated differentially methylated region; CGI: CpG island; ChIP-seq: chromatin immunoprecipitation; ESC: embryonic stem cells; FPKM: fragments per kilobase of exon per million fragments mapped; iiDMR: inter-individual differentially methylated region; MZ: monozygotic; PBMC: peripheral blood mononuclear cells; RMS: root mean square; SNEP: single nucleosome epi-polymorphism; SNP: single nucleotide polymorphism; TSS: transcriptional start site.

## Competing interests

The authors declare that they have no competing interests.

## Authors' contributions

CG performed the majority of the experimental work and helped to draft the manuscript. TAD performed the bioinformatics analyses. HB and MIH collected and processed blood samples from the MZ twin pair participants. MLH assisted CG with various aspects of the experimental work. PJH assisted with the ChIP-seq. GG, RDL and GCE contributed reagents and materials. SVR and VKR conceived and designed the study, participated in its design, coordination and analysis, and helped to draft the manuscript. All authors read and approved the final manuscript.

## Accession codes

All data are available on GEO [GSE46220].

## Supplementary Material

Additional file 1 **Additional Materials and methods, Tables S1 and S2, and Figures S1 to S5**. Click here for file

## References

[B1] Richards EJ Population epigenetics. Curr Opin Genet Dev 2008 14 221 226 10.1016/j.gde.2008.01.014 18337082

[B2] Miura K Agetsuma M Kitano H Yoshimura A Matsuoka M Jacobsen SE Ashikari M A metastable DWARF1 epigenetic mutant affecting plant stature in rice. Proc Natl Acad Sci USA 2009 14 11218 11223 10.1073/pnas.0901942106 19541604 PMC2708680

[B3] Schmitz RJ Schultz MD Lewsey MG O'Malley RC Urich MA Libiger O Schork NJ Ecker JR Transgenerational epigenetic instability is a source of novel methylation variants. Science 2011 14 369 373 10.1126/science.1212959 21921155 PMC3210014

[B4] Becker C Hagmann J Müller J Koenig D Stegle O Borgwardt K Weigel D Spontaneous epigenetic variation in the *Arabidopsis thaliana *methylome. Nature 2011 14 245 249 10.1038/nature10555 22057020

[B5] Rakyan VK Down TA Balding DJ Beck S Epigenome-wide association studies for common human diseases. Nat Rev Genet 2011 14 529 541 10.1038/nrg3000 21747404 PMC3508712

[B6] Zhang D Cheng L Badner JA Chen C Chen Q Luo W Craig DW Redman M Gershon ES Liu C Genetic control of individual differences in gene-specific methylation in human brain. Am J Hum Genet 2010 14 411 419 10.1016/j.ajhg.2010.02.005 20215007 PMC2833385

[B7] Bell JT Pai AA Pickrell JK Gaffney DJ Pique-Regi R Degner JF Gilad Y Pritchard JK DNA methylation patterns associate with genetic and gene expression variation in HapMap cell lines. Genome Biol 2011 14 R10 R16 10.1186/gb-2011-12-1-r10 21251332 PMC3091299

[B8] Kaminsky ZA Tang T Wang SC Ptak C Oh GH Wong AH Feldcamp LA Virtanen C Halfvarson J Tysk C McRae AF Visscher PM Montgomery GW Gottesman II Martin NG Petronis A DNA methylation profiles in monozygotic and dizygotic twins. Nature Genet 2009 14 240 245 10.1038/ng.286 19151718

[B9] Fraga MF Ballestar E Paz MF Ropero S Setien F Ballestar ML Heine-Suñer D Cigudosa JC Urioste M Benitez J Boix-Chornet M Sanchez-Aguilera A Ling C Carlsson E Poulsen P Vaag A Stephan Z Spector TD Wu YZ Plass C Esteller M Epigenetic differences arise during the lifetime of monozygotic twins. Proc Natl Acad Sci USA 2005 14 10604 10609 10.1073/pnas.0500398102 16009939 PMC1174919

[B10] Sandovici I Smith NH Nitert MD Ackers-Johnson M Uribe-Lewis S Ito Y Jones RH Marquez VE Cairns W Tadayyon M O'Neill LP Murrell A Ling C Constância M Ozanne SE Maternal diet and aging alter the epigenetic control of a promoter-enhancer interaction at the Hnf4a gene in rat pancreatic islets. Proc Natl Acad Sci USA 2011 14 5449 5454 10.1073/pnas.1019007108 21385945 PMC3069181

[B11] Waterland RA Kellermayer R Laritsky E Rayco-Solon P Harris RA Travisano M Zhang W Torskaya MS Zhang J Shen L Manary MJ Prentice AM Season of conception in rural Gambia affects DNA methylation at putative human metastable epialleles. PLoS Genet 2011 14 e1001252 10.1371/journal.pgen.1001252 PMC3009670 21203497

[B12] Hoile SP Lillycrop KA Thomas NA Hanson MA Burdge GC Dietary protein restriction during F0 pregnancy in rats induces transgenerational changes in the hepatic transcriptome in female offspring. PLoS One 2011 14 e21668 10.1371/journal.pone.0021668 21750721 PMC3131279

[B13] Breitling LP Yang R Korn B Burwinkel B Brenner H Tobacco-smoking-related differential DNA methylation: 27K discovery and replication. Am J Hum Genet 2011 14 450 457 10.1016/j.ajhg.2011.03.003 21457905 PMC3071918

[B14] Bird A Putting the DNA back into DNA methylation. Nat Genet 2011 14 1050 1051 10.1038/ng.987 22030606

[B15] Sandoval J Heyn H Moran S Serra-Musach J Pujana MA Bibikova M Esteller M Validation of a DNA methylation microarray for 450,000 CpG sites in the human genome. Epigenetics 2011 14 692 702 10.4161/epi.6.6.16196 21593595

[B16] Conerly ML Teves SS Diolaiti D Ulrich M Eisenman RN Henikoff S Changes in H2A.Z occupancy and DNA methylation during B-cell lymphomagenesis. Genome Res 2010 14 1383 1390 10.1101/gr.106542.110 20709945 PMC2945187

[B17] Rakyan VK Beyan H Down TA Hawa MI Maslau S Aden D Daunay A Busato F Mein CA Manfras B Dias KR Bell CG Tost J Boehm BO Beck S Leslie RD Identification of type 1 diabetes-associated DNA methylation variable positions that precede disease diagnosis. PLoS Genet 2011 14 e1002300 10.1371/journal.pgen.1002300 21980303 PMC3183089

[B18] Feinberg AP Irizarry RA Fradin D Aryee MJ Murakami P Aspelund T Eiriksdottir G Harris TB Launer L Gudnason V Fallin MD Personalized epigenomic signatures that are stable over time and covary with body mass index. Sci Transl Med 2010 14 49ra67 10.1126/scitranslmed.3001262 20844285 PMC3137242

[B19] Finer S Holland ML Nanty L Rakyan VK The hunt for the epiallele. Environ Mol Mutagen 2011 14 1 11 10.1002/em.20590 20839222

[B20] Rakyan VK Down TA Maslau S Andrew T Yang TP Beyan H Whittaker P McCann OT Finer S Valdes AM Leslie RD Deloukas P Spector TD Human aging-associated DNA hypermethylation occurs preferentially at bivalent chromatin domains. Genome Res 2010 14 434 439 10.1101/gr.103101.109 20219945 PMC2847746

[B21] Teschendorff AE Menon U Gentry-Maharaj A Ramus SJ Weisenberger DJ Shen H Campan M Noushmehr H Bell CG Maxwell AP Savage DA Mueller-Holzner E Marth C Kocjan G Gayther SA Jones A Beck S Wagner W Laird PW Jacobs IJ Widschwendter M Age-dependent DNA methylation of genes that are suppressed in stem cells is a hallmark of cancer. Genome Res 2010 14 440 446 10.1101/gr.103606.109 20219944 PMC2847747

[B22] Bernstein BE Mikkelsen TS Xie X Kamal M Huebert DJ Cuff J Fry B Meissner A Wernig M Plath K Jaenisch R Wagschal A Feil R Schreiber SL Lander ES A bivalent chromatin structure marks key developmental genes in embryonic stem cells. Cell 2006 14 315 326 10.1016/j.cell.2006.02.041 16630819

[B23] Azuara V Perry P Sauer S Spivakov M Jørgensen HF John RM Gouti M Casanova M Warnes G Merkenschlager M Fisher AG Chromatin signatures of pluripotent cell lines. Nat Cell Biol 2006 14 532 538 10.1038/ncb1403 16570078

[B24] Zhao XD Han X Chew JL Liu J Chiu KP Choo A Orlov YL Sung WK Shahab A Kuznetsov VA Bourque G Oh S Ruan Y Ng HH Wei CL Whole-genome mapping of histone H3 Lys4 and 27 trimethylations reveals distinct genomic compartments in human embryonic stem cells. Cell Stem Cell 2007 14 286 298 10.1016/j.stem.2007.08.004 18371363

[B25] Gertz J Varley KE Reddy TE Bowling KM Pauli F Parker SL Kucera KS Willard HF Myers RM Analysis of DNA methylation in a three-generation family reveals widespread genetic influence on epigenetic regulation. PLoS Genet 2011 14 e1002228 10.1371/journal.pgen.1002228 21852959 PMC3154961

[B26] Nica AC Parts L Glass D Nisbet J Barrett A Sekowska M Travers M Potter S Grundberg E Small K Hedman AK Bataille V Tzenova Bell J Surdulescu G Dimas AS Ingle C Nestle FO di Meglio P Min JL Wilk A Hammond CJ Hassanali N Yang TP Montgomery SB O'Rahilly S Lindgren CM Zondervan KT Soranzo N Barroso I Durbin R The architecture of gene regulatory variation across multiple human tissues: the MuTHER study. PLoS Genet 2011 14 e1002003 10.1371/journal.pgen.1002003 21304890 PMC3033383

[B27] Nagarajan M Veyrieras JB de Dieuleveult M Bottin H Fehrmann S Abraham AL Croze S Steinmetz LM Gidrol X Yvert G Natural single-nucleosome epi-polymorphisms in yeast. PLoS Genet 2010 14 e1000913 10.1371/journal.pgen.1000913 20421933 PMC2858693

[B28] Parts L Hedman ÅK Keildson S Knights AJ Abreu-Goodger C van de Bunt M Guerra-Assunção JA Bartonicek N van Dongen S Mägi R Nisbet J Barrett A Rantalainen M Nica AC Quail MA Small KS Glass D Enright AJ Winn J MuTHER Consortium Deloukas P Dermitzakis ET McCarthy MI Spector TD Durbin R Lindgren CM Extent, causes, and consequences of small RNA expression variation in human adipose tissue. PLoS Genet 2012 14 e1002704 10.1371/journal.pgen.1002704 22589741 PMC3349731

[B29] Teschendorff AE Jones A Fiegl H Sargent A Zhuang JJ Kitchener HC Widschwendter M Epigenetic variability in cells of normal cytology is associated with the risk of future morphological transformation. Genome Med 2012 14 24 10.1186/gm323 22453031 PMC3446274

[B30] Meissner A Mikkelsen TS Gu H Wernig M Hanna J Sivachenko A Zhang X Bernstein BE Nusbaum C Jaffe DB Gnirke A Jaenisch R Lander ES Genome-scale DNA methylation maps of pluripotent and differentiated cells. Nature 2008 14 766 770 18600261 10.1038/nature07107 PMC2896277

[B31] Ohm JE McGarvey KM Yu X Cheng L Schuebel KE Cope L Mohammad HP Chen W Daniel VC Yu W Berman DM Jenuwein T Pruitt K Sharkis SJ Watkins DN Herman JG Baylin SB A stem cell-like chromatin pattern may predispose tumor suppressor genes to DNA hypermethylation and heritable silencing. Nat Genet 2007 14 237 242 10.1038/ng1972 17211412 PMC2744394

[B32] Schlesinger Y Straussman R Keshet I Farkash S Hecht M Zimmerman J Eden E Yakhini Z Ben-Shushan E Reubinoff BE Bergman Y Simon I Cedar H Polycomb-mediated methylation on Lys27 of histone H3 pre-marks genes for de novo methylation in cancer. Nat Genet 2007 14 232 236 10.1038/ng1950 17200670

[B33] Widschwendter M Fiegl H Egle D Mueller-Holzner E Spizzo G Marth C Weisenberger DJ Campan M Young J Jacobs I Laird PW Epigenetic stem cell signature in cancer. Nat Genet 2007 14 157 158 10.1038/ng1941 17200673

[B34] Cuddapah S Jothi R Schones DE Roh TY Cui K Zhao K Global analysis of the insulator binding protein CTCF in chromatin barrier regions reveals demarcation of active and repressive domains. Genome Res 2009 14 4 32 10.1101/gr.082800.108 PMC2612964 19056695

