# Supplementary material for: Inactive or moderately active human promoters are enriched for inter-individual epialleles
Source: Genome Biol. 2013 May 25;14(5):R43. doi: 10.1186/gb-2013-14-5-r43 (PMC4053860; doi:10.1186/gb-2013-14-5-r43)
Supplement: Additional file 1 — Additional Materials and methods, Tables S1 and S2, and Figures S1 to S5. [file gb-2013-14-5-r43-S1.DOCX]

**Supplementary Materials and Methods for:**

**Inactive or moderately active human promoters are enriched for inter-individual epialleles**

**Carolina Gemma^1,5^, Sreeram V Ramagopalan^1,2,5^, Thomas A Down^3,5^, Huriya Beyan^1,5^, Mohammed I. Hawa^1^, Michelle L Holland^1^, Paul J Hurd^4^**, **Gavin Giovannoni^1^, R David Leslie^1^, George C Ebers^5^ & Vardhman K Rakyan^1^**

^1^ The Blizard Institute, Barts and The London School of Medicine and Dentistry, Queen Mary University of London, 4 Newark Street, London E1 2AT, UK.

^2^ Department of Physiology, Anatomy and Genetics and Medical Research Council Functional Genomics Unit, South Parks Road, Oxford, OX1 3PT, UK

^3^ The Gurdon Institute and Department of Genetics, University of Cambridge, Tennis Court Road, Cambridge CB2 1QN, UK.

^4^ School of Biological and Chemical Sciences, Queen Mary University of London, London, E1 4NS, UK.

^5^ Wellcome Trust Centre for Human Genetics, University of Oxford, Headington, Oxford OX3 7BN, UK.

^6^ These authors contributed equally to this work.

Correspondence should be addressed to VKR ([v.rakyan@qmul.ac.uk](mailto:v.rakyan@qmul.ac.uk)) or SVR ([s.ramagopalan@qmul.ac.uk](mailto:s.ramagopalan@qmul.ac.uk))

**Supplementary Methods**

**ChIP-seq.** 5 x 10^5^ CD14+ cells were crosslinked with 1% formaldehyde in RPMI for 8 min at RT before addition of glycine to a final concentration of 0.125 M. After washing twice with ice-cold PBS containing 1X protease inhibitor cocktail (PIC) (Sigma) cells were resuspended in 10 ml ice-cold cell lysis buffer 1 (10mM Tris-CL pH 8.0, 10mM EDTA, 10mM Na-Butyrate, 0.5mM EGTA, 0.25% Triton X-100, 1X PIC, 1 mM PMSF) and incubated on ice 10 min. Nuclei were recovered by centrifugation and resuspended in 10 ml lysis buffer 2 (0.2M NaCl, 10mM Tris-CL pH 8.0, 1mM EDTA, 0.5mM EGTA, 1X PIC, 1 mM PMSF) and incubated 10 min. Samples were centrifuged and resuspended in 1 ml sonication buffer (50mM Hepes pH 7.9, 140 mM NaCl, 1 mM EDTA, 1% Triton X-100, 0.1% Na-deoxycholate, 0.1% SDS, 1 mM PMSF ). Chromatin was sonicated for 30 min on ice using a Jencson probe sonicator (Fisher Scientific) at 20% power, alternating 30 seconds on and 30 seconds off to give 100-500 bp. The soluble chromatin was then separated from the cellular debris by centrifugation and used for immunoprecipitation, 10% of the chromatin was separated as input.

For the immunoprecipitation 25 µl of Dynabeads Protein A (Dynal Biotech ASA, Oslo, Norway Invitrogen cat no: 100.01D), were incubated for 40 min at RT with 10 ul of anti-H2A.Z antibody (active motif, Cat no: 39113) After 2 washes with PBS to remove unbound antibody, the chromatin was added to the antibody-bead complexes and incubated overnight at 4 C with slow rotation. After successive washes with RIPA buffer (10 mM Tris-HCl (pH 7.6), 1mM EDTA, 0.1% SDS, 0.1% sodium deoxycholate, 1% Triton X-100), RIPA buffer+0.3M NaCl, LiCl buffer (0.25 M LiCl, 0.5% NP-40, 0.5% NaDOC), TE + 0.2% Triton X-100, the complexes were separated and reverse cross-linked by incubation with proteinase K at 65 C ON and the DNA purified with Qiaquick PCR purification kit (Qiagen, cat. No: 28106). ChIP-seq libraries were prepared following the Illumina protocol and ligated to standard PE adaptors and sequenced on an Illumina GAIIx instrument. One lane of 72bp paired-end sequencing was performed for each ChIP-seq library.

**RNA-seq.** Total RNA was isolated from CD14+ cells using the Qiagen RNeasy kit. RNA-seq libraries were prepared using the TruSeq RNA kit from Illumina using 200 ng of total RNA from CD14+ cells. Poly-T-oligo-attached magnetic beads were used to purify the mRNA fraction which was then fragmented. First strand cDNA synthesis was performed using random primers and SuperScript II (Invitrogen, cat. No: 18064-014). Then double stranded cDNA was generated and used as a template for adaptor ligation using TruSeq adaptors. RNA-seq libraries were sequenced on an Illumina GAIIx instrument, 1 lane of 36 bp paired-end sequencing was performed for each sample.

**Supplementary figures and tables**

**Table S1.** Monozygotic twin volunteers who participated in this study.

11: Male, DOB - 24/02/86

21: Female, DOB - 26/06/80

22: Female, DOB - 26/06/80

31: Female, DOB - 05/10/78

32: Female, DOB - 05/10/78

All are of white European origin, and negative to GADA, IA2 and ZnT8 autoantibodies

**Table S2.** Comparison of genetic variation among the five different individuals in the discovery set.

Number of SNPs between:

31 v 32 – 104

21 v 22 – 115

31 v 21 – 648,960

31 v 11 – 653,093

11 v 22 – 657,821


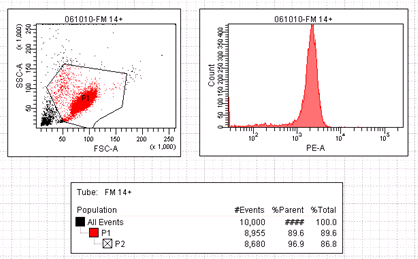


**Figure S1** Purity of CD14+ cells determined by FACS using CD14-FITC antibodies.

**Figure S2.** For the CpGs in common between the second (450K array) and first time-points (27K arrays), shown is the proportion of methylation differences found at the second time-point that were also present at the first time-point.

**Figure S3.** Log10 fold differences in H2A.z ChIP signal for all iiDMRs located in promoter regions (Ensembl TSS +/- 1KB).


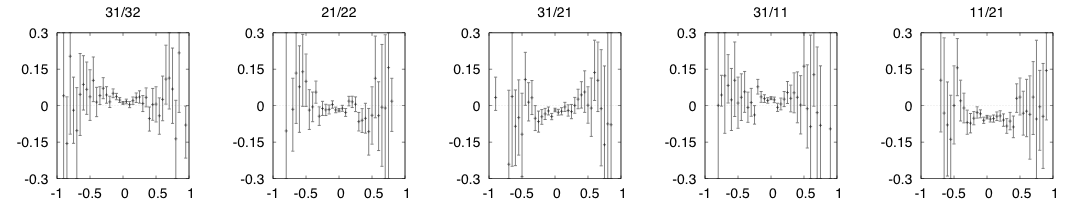


**Figure S4.** Relation between promoter H2A.Z differences and expression differences. For each bin of H2A.z log-ratios, we calculated (FPKM) log-ratio and show 95% credible intervals on the mean

**Figure S5.** For this analysis we used 30 MZ twin pairs from Ref. 20 of the main text whose whole blood DNA methylation profiles were generated by Illumina 27K arrays. We defined low and high expression based on whole blood expression data from (GNF Atlas). High expression: > 10 normalized expression signal, Low expression <10 normalized expression signal.
